# Specific calpain inhibition protects kidney against inflammaging

**DOI:** 10.1038/s41598-017-07922-1

**Published:** 2017-08-14

**Authors:** Guillaume Hanouna, Laurent Mesnard, Sophie Vandermeersch, Joëlle Perez, Sandrine Placier, Jean-Philippe Haymann, Fabien Campagne, Julien Moroch, Aurélien Bataille, Laurent Baud, Emmanuel Letavernier

**Affiliations:** 1 Sorbonne Universités, UPMC Univ Paris 06, UMR S 1155, F-75020 Paris, France; 2 INSERM, UMR S 1155, F-75020 Paris, France; 3 Physiology Unit, AP-HP, Hôpital Tenon, F-75020 Paris, France; 4 The HRH Prince Alwaleed Bin Talal Bin Abdulaziz Alsaud Institute for Computational Biomedicine, Weill Cornell Medical College, New York, United States of America; 5 Department of Physiology and Biophysics, The Weill Cornell Medical College, New York, United States of America; 6 Pathology Unit, AP-HP, Hôpital Tenon, F-75020 Paris, France

## Abstract

Calpains are ubiquitous pro-inflammatory proteases, whose activity is controlled by calpastatin, their specific inhibitor. Transgenic mice over-expressing rabbit calpastatin (CalpTG) are protected against vascular remodelling and angiotensin II-dependent inflammation. We hypothesized that specific calpain inhibition would protect against aging-related lesions in arteries and kidneys. We analysed tissues from 2-months and 2-years-old CalpTG and wild-type mice and performed high throughput RNA-Sequencing of kidney tissue in aged mice. In addition, we analysed inflammatory response in the kidney of aged CalpTG and wild-type mice, and in both *in vivo* (monosodium urate peritonitis) and *in vitro* models of inflammation. At two years, CalpTG mice had preserved kidney tissue, less vascular remodelling and less markers of senescence than wild-type mice. Nevertheless, CalpTG mice lifespan was not extended, due to the development of lethal spleen tumors. Inflammatory pathways were less expressed in aged CalpTG mice, especially cytokines related to NF-κB and NLRP3 inflammasome activation. CalpTG mice had reduced macrophage infiltration with aging and CalpTG mice produced less IL-1α and IL-1β *in vivo* in response to inflammasome activators. *In vitro*, macrophages from CalpTG mice produced less IL-1α in response to particulate activators of inflammasome. Calpains inhibition protects against inflammaging, limiting kidney and vascular lesions related to aging.

## Introduction

Calpains 1 and 2 (or µ and m) are ubiquitous calcium-activated proteases involved in pathological cardiovascular remodelling and development of inflammatory kidney diseases. Both proteases share many similar substrates and their activity is blunted by calpastatin, their natural, ubiquitous and specific inhibitor^1^. Since the deletion of both calpains 1 and 2 is lethal, we previously developed transgenic mice overexpressing rabbit calpastatin, which contains a substrate-like inhibitory sequence similar to mouse calpastatin (CalpTG mice)^2^. These mice have normal baseline calpain activity and phenotype and the increase in calpastatin expression has been shown previously to blunt specifically calpain activation in models of glomerulonephritis, sepsis, ischemia or pathological neo-angiogenesis^2–5^. Unlike synthetic inhibitors, calpastatin inhibits selectively calpains and not other proteases. Calpains promote inflammation onset through several mechanisms. They degrade IκBα on a specific PEST sequence, leading to NF-κB activation and pro-inflammatory cytokines production^1, 6^. In addition to cytokine transcription, calpains promote interleukin-1α (IL-1α) maturation and activation^7^. Calpains also limit glucocorticoid anti-inflammatory activity by degrading HSP-90 and are essential for inflammatory cell recruitment, migration and diapedesis^1, 8, 9^. Accordingly, calpastatin overexpression limits NF-κB activation and inflammatory cell migration^10, 11^.

We previously described that calpains promote the development of inflammatory and fibrotic lesions in kidney and arteries in response to angiotensin II, and that calpastatin overexpression protected mice against these lesions^10^. Interestingly, angiotensin II, through AT-1 receptor signalling, promotes vascular aging^12^. Moreover, calpains are potent mediators of neurological diseases, including Alzheimer disease^13, 14^. A specific feature of tissue aging is the development of an inflammatory phenotype, including overexpression of innate immunity and inflammasome-related cytokines, so-called “inflammaging”^15^. Although its expression is weak and difficult to assess *in vivo*, one of the main cytokine linked to inflammaging is interleukin-1α (IL-1α)^16^. It was therefore tempting to speculate that calpains would promote vascular and kidney aging. At last, proteomic analysis performed in Klotho-invalidated mice revealed that in the absence of Klotho, the main anti-aging mechanism identified to date, alpha-spectrin clivage was strongly increased. Further analyses identified that this clivage was specific to calpains, evidencing thereby that Klotho may decrease calpain activity^17^.

We hypothesized that calpains would promote aging and especially kidney and cardiovascular lesions related to aging. We show hereafter that specific calpain inhibition by calpastatin overexpression decreases tissue inflammation and kidney and vascular lesions related to aging.

## Results

### Evolution of kidney calpain expression and calpain activity with aging in control (WT) and CalpTG mice

**Figure 1:**
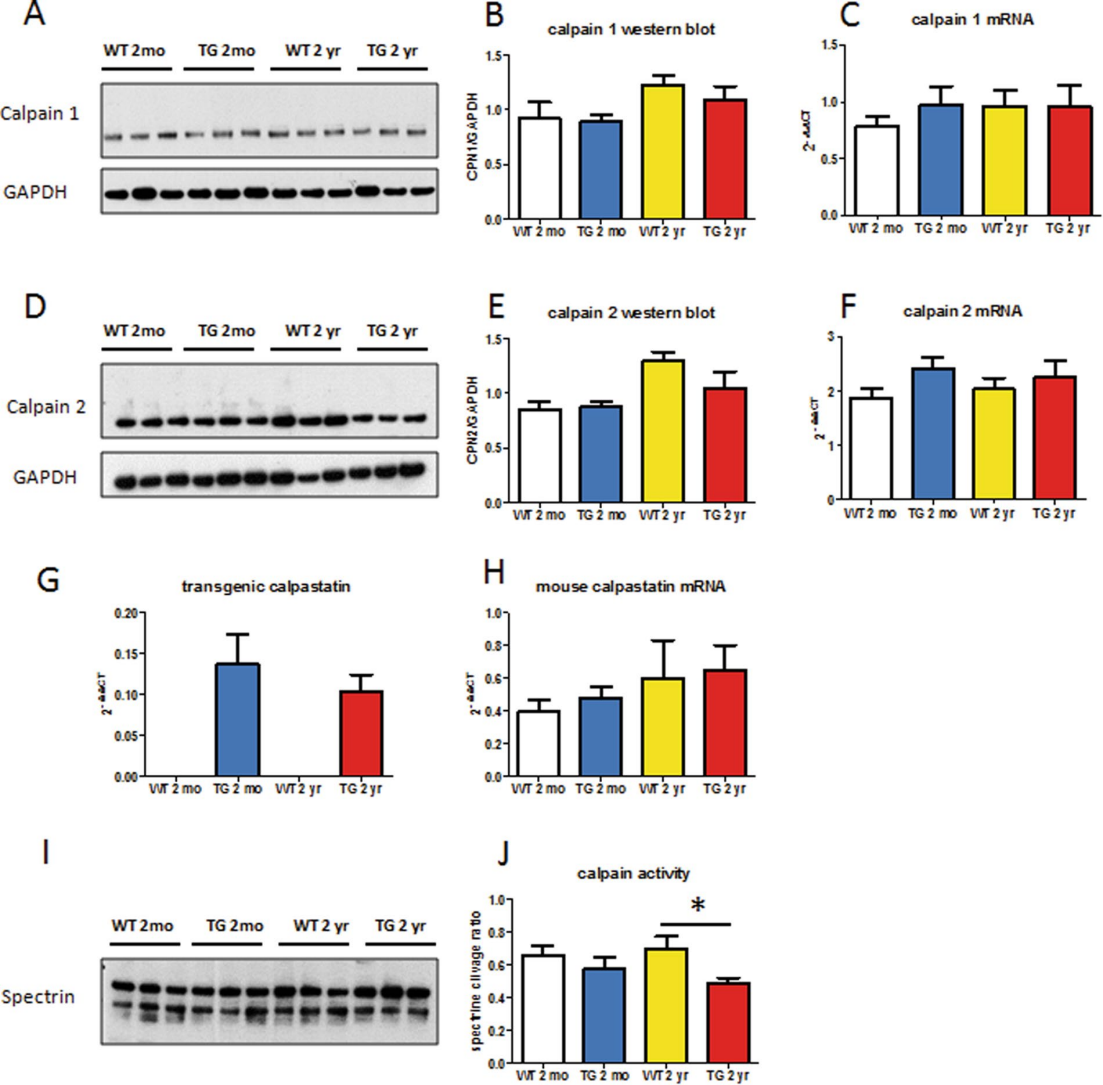
Calpastatin overexpression decreases calpain activity in aged mice. Western blots and quantitative PCR have been performed by using kidney cortex extracts from 2-months old (2mo) and 2 years old (2 yr) wild type (WT) and CalpTG (TG) mice. There was no significant difference of calpain 1 expression in WT and CalpTG mice at 2 months or 2 years at the protein level (**A,B**, n = 6/group) or at the RNA level (**C**, n = 6/group). Similarly, calpain 2 expression did not differ significantly between the 2 groups at the protein level (**D,E**, n = 6/group) or at the mRNA level (**F**, n = 6/group). As expected and previously described, only CalpTG mice expressed the transgenic rabbit calpastatin (**G**, n = 6/group). Mouse calpastatin mRNA expression did not differ among the different groups (**H**, n = 6/group). Calpain activity was measured by the ratio of the 145 kDa specific breakdown product of spectrin A by calpains, indexed to the intact spectrin. Calpains activity was similar at 2 months but significantly decreased in 2-years old CalpTG mice in comparison to aged WT mice (**I**,**J**, *p < 0.05, n = 6/group).

To assess the evolution of calpain expression with aging in kidney cortex, we measured protein and mRNA expression of calpain 1 and 2 in CalpTG and WT mice (Fig. 1A–F). There was no significant modification of calpain expression with aging (Fig. 1A–F). As expected and previously described^2–5, 9–11^, only CalpTG mice expressed the calpastatin transgene (Fig. 1G–H).

Calpain activity has been assessed *in vivo* in kidney cortex by measuring alpha-spectrin cleavage and the production of the 145 kDa specific breakdown product, as usually performed (2–5,10). Calpain activity was significantly lower in kidneys from CalpTG mice at 2 years when compared to WT mice (Fig. 1I–J, p = 0.041).

### Development of aging-related lesions in WT and CalpTG mice kidneys

**Figure 2:**
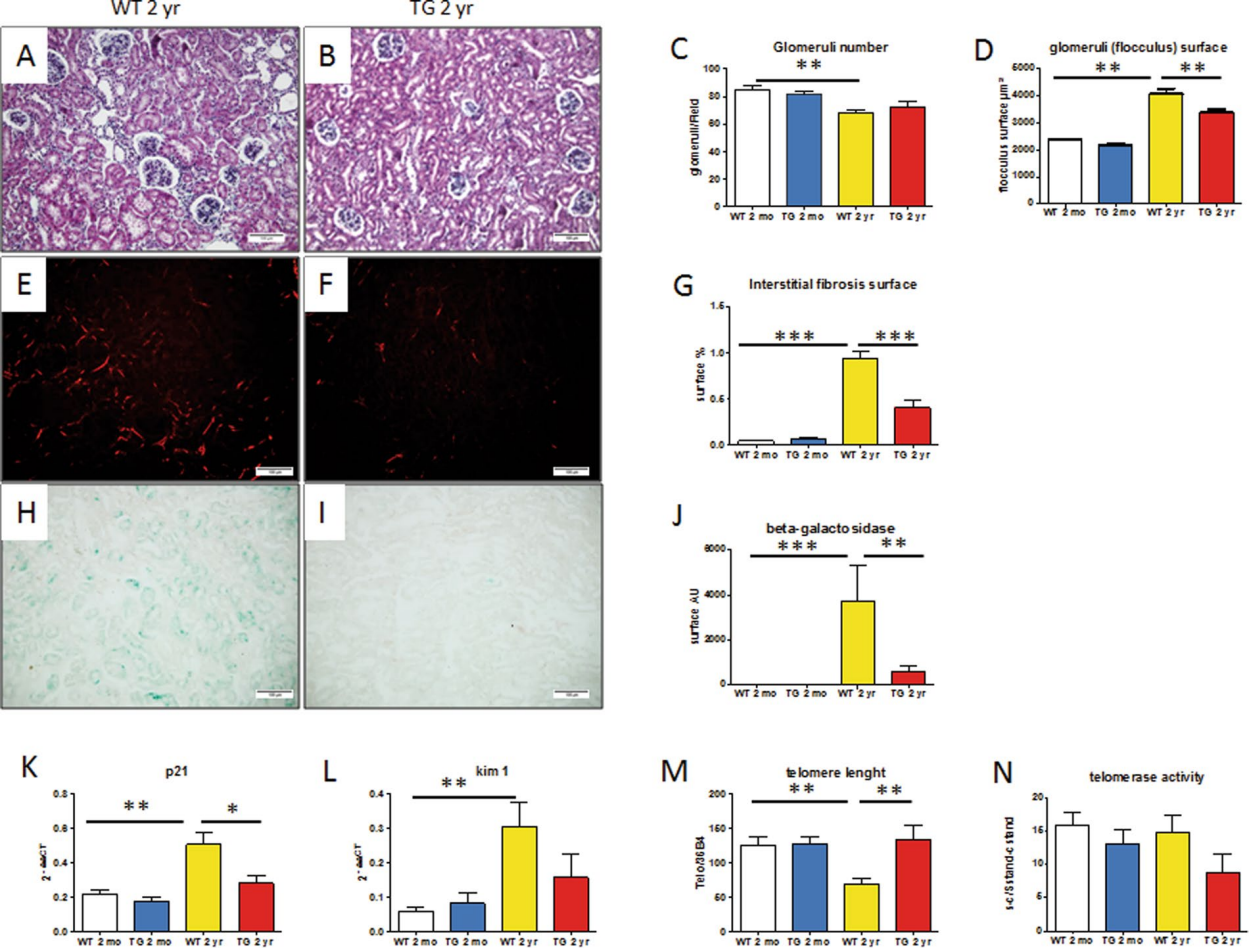
Calpastatin overexpression protects against kidney aging. At 2 years, CalpTG (TG) mice kidney was less impacted by aging than WT mice bred in the same conditions (**A,B**). The number of glomeruli/field decreased in WT animals at 2 years (**C**, *p < 0.05, n = 10/group). By contrast, the number of glomeruli did not decrease significantly in CalpTG animals. At 2 years, kidneys were affected by glomerulosclerosis and enlarged glomeruli in WT animals, but these lesions were significantly less important in CalpTG mice (**D**, **p < 0.01, n = 10/group, magnification ×200). Fibrosis quantification was assessed by sirius red morphometry under polarized light, revealing that at 2 years, interstitial fibrosis surface was reduced in CalpTG mice when compared to aged WT (**E–G**, ***p < 0.001, n = 10/group, magnification ×200). Beta-galactosidase activity, a marker of senescence was dramatically reduced at 2 years in CalpTG mice (**H–J**, *p < 0.05, n = 5/group, magnification ×200). p21, another classical marker of senescence was also less expressed in CalpTG animals at 2 years (**K**, *p < 0.05, n = 6/group). Kim-1, a marker of tubular injury increased significantly with aging only in WT mice (**L**, **p < 0.01, n = 6/group). Telomere shortening was significantly more important in WT mice at 2 years in comparison to CalpTG animals (**M**, **p < 0.01, n = 10/group). The protection of telomeres in CalpTG mice could not be ascribed to increased telomerase activity (N, n = 8/group).

Renal histology has been analysed in young and aged WT and CalpTG mice (Fig. 2A–D). Aging was characterized by a significant decrease in the number of glomeruli in WT mice only (Fig. 2C, p = 0.0022). At 2 years, CalpTG mice developed less glomerulosclerosis than WT mice (Fig. 2D, p = 0.0085). CalpTG mice were also protected against the development of interstitial fibrosis at 2 years (Fig. 2E–G, p = 0.0005).

CalpTG mice had a reduced beta-galactosidase activity, a marker of senescence in response to oxidative stress, in tubular cells at 2 years (Fig. 2H–J, p = 0.031). The expression of p21, another marker of senescence was also reduced in aged CalpTG mice (Fig. 2K, p = 0.01). Kim-1 expression, a marker of tubular injury was significantly increased with aging in WT mice but not in CalpTG mice kidney cortex (Fig. 2L, p = 0.0043).

**Figure 3:**
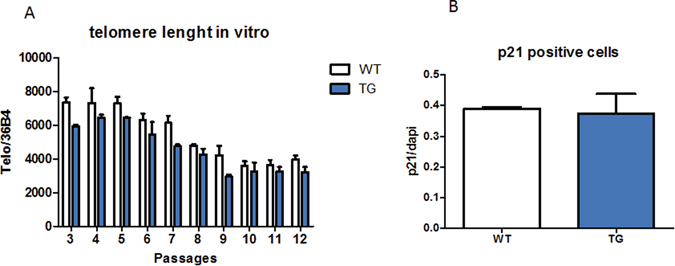
Calpastatin overexpression in tubular cells in vitro does not protect against aging. Prominin 1 positive proximal tubular cells have been isolated by flow cytometry and cultured during 5 months. After 12 passages, telomere lenght decreased similarly in cells from WT and CalpTG (TG) mice (**A**, n = 3). In addition, after 6 passages, a similar proportion of WT and CalpTG cells expressed p21, a classical marker of senescence (**B**, n = 3).

The telomere lenght was also preserved in aged CalpTG mice in comparison to aged WT animals (Fig. 2M, p = 0.007). This could not be ascribed to an increased telomerase activity (Fig. 2N). To assess whether calpastatin transgene could by itself protect against replicative senescence, a culture of tubular cells from CalpTG and WT mice kidneys has been performed during 5 months. There was no difference in telomere length shortening or p21 expression *in vitro*, evidencing that the differences of telomere shortening observed *in vivo* result from cellular interactions with the environment, e.g. oxydative stress (Fig. 3A,B).

### Development of cardiovascular remodelling with aging in WT and CalpTG mice

**Figure 4:**
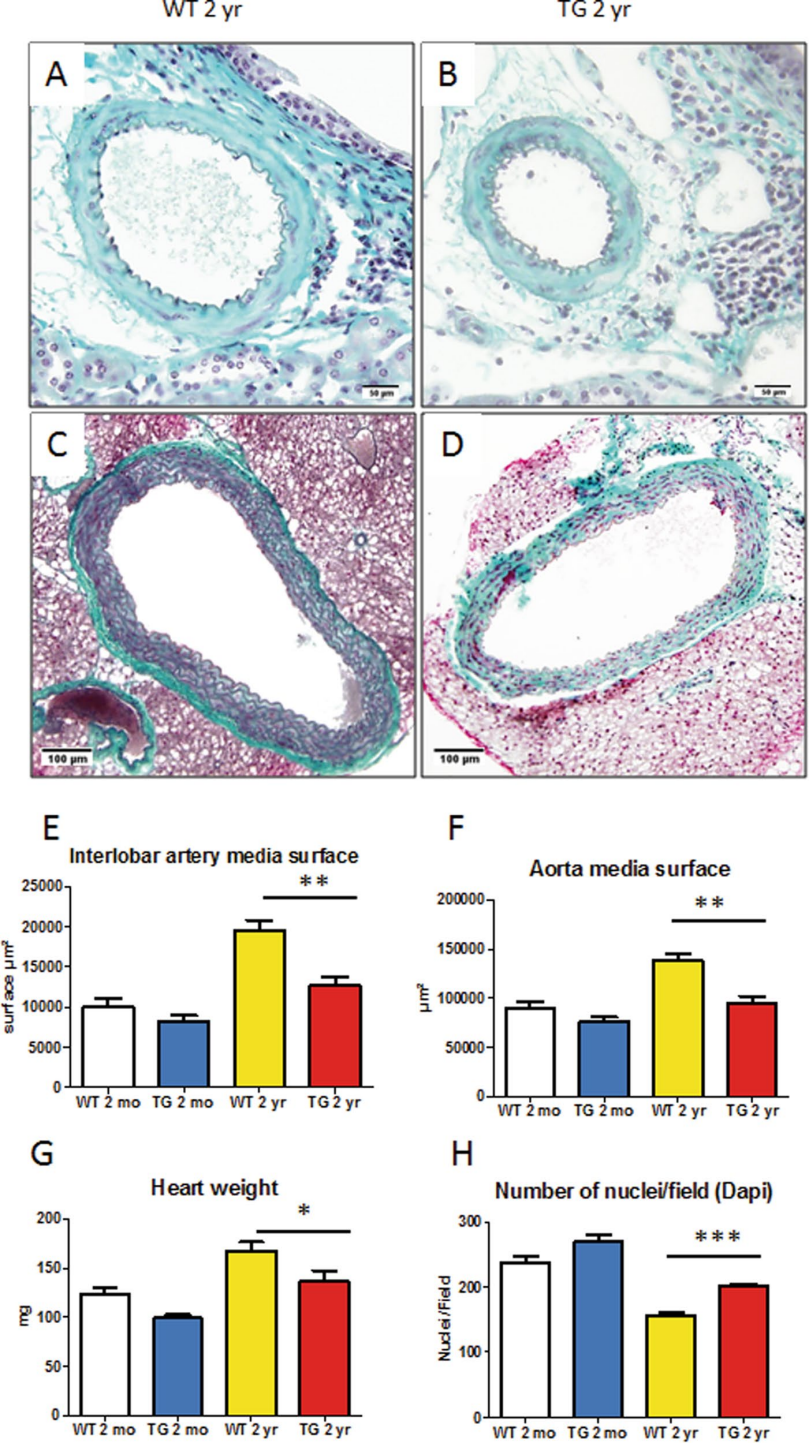
Calpastatin overexpression protects against cardiovascular aging. Mean media surface of interlobar arteries was significantly less increased in CalpTG (TG) mice at 2 years in comparison to WT mice (**A,B,E**, **p < 0.01, n = 10/group, magnification ×400). Similarly, aorta remodelling was less important in CalpTG mice (**C,D,F**, **p < 0.01, n = 10/group, magnification ×200). Heart weight increased with aging but less in CalpTG mice than in WT animals (**G**, *p < 0.05, n = 10/group). This increase in heart weight was due to cellular hypertrophy since the number of nuclei per field at 400x magnification was significantly lower in WT in comparison to CalpTG mice (**H**, ***p < 0.001, n = 5/group).

Aging was associated with the medial wall thickening of renal interlobar arteries, that was in a large part prevented in CalpTG mice (Fig. 4A,B,E, p = 0.0015). Similarly, aorta medial wall surface was less increased with aging in CalpTG mice, in contrast with WT mice (Fig. 4C,D,F, p = 0.0011). CalpTG mice were also protected against the heart remodelling that occurs with aging (Fig. 4G, p = 0.015). The modifications in heart weight were actually due to cardiomyocyte hypertrophy since morphometric analyses evidenced that the number of cardiomyocyte nuclei/surface decreased with aging, but to a lower extent in CalpTG mice (Fig. 4H, p = 0.0002).

**Figure 5:**
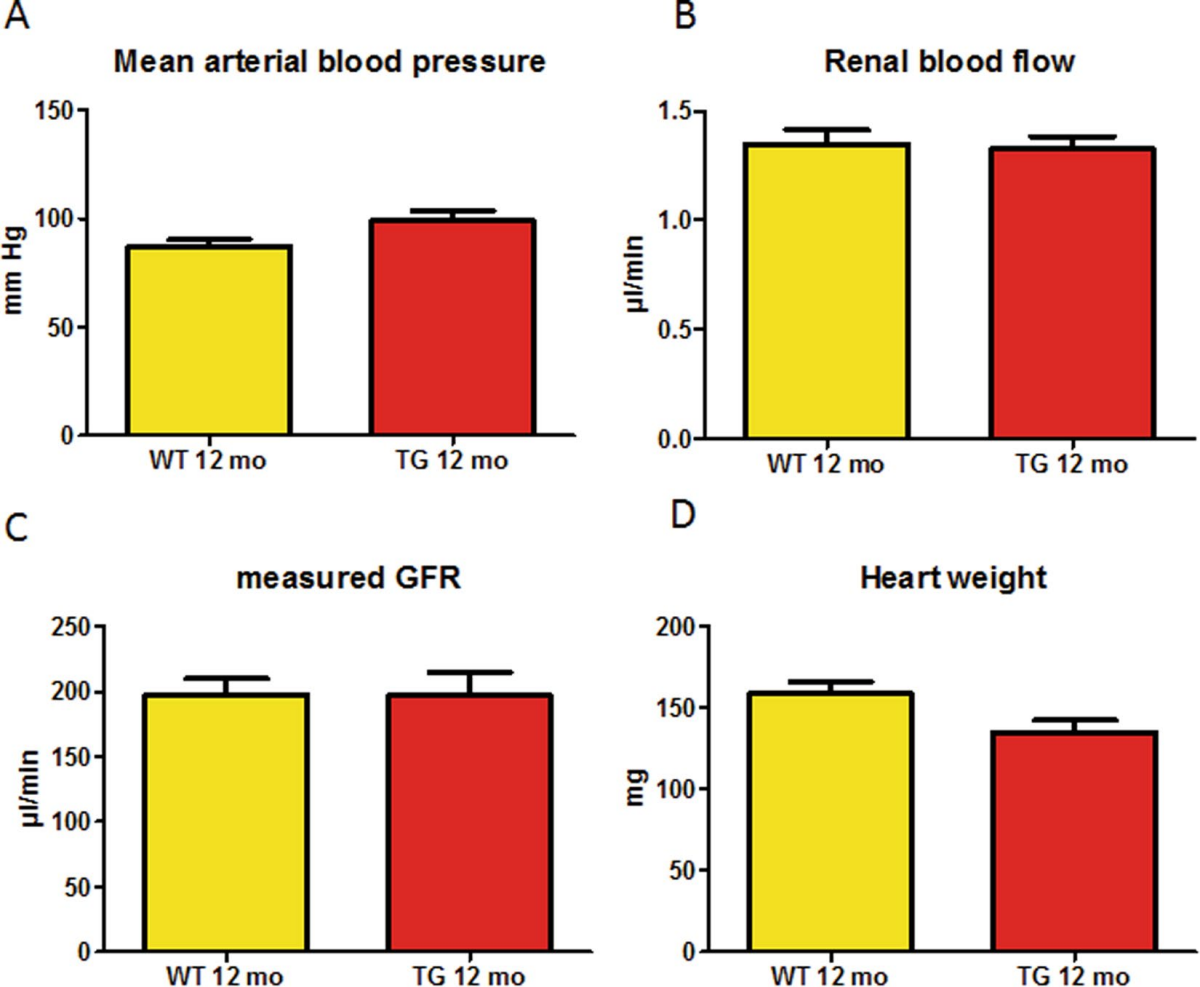
Calpastatin overexpression does not impact hemodynamic or renal blood flow. At one year, blood pressure, renal blood flow and glomerular filtration rate (GFR) were similar in CalpTG (TG) and WT mice, evidencing that calpastatin does not protect kidney through hemodynamic improvement (**A–C**, n = 6–10/group). Although hemodynamic features were similar, there was a trend toward lower heart mass in CalpTG (TG) mice at one year (**D**, p = 0.05, n = 7/group).

To analyse whether calpastatin protection against cardiovascular remodelling or even kidney lesions would result from changes in hemodynamics, a specific set of invasive experiments has been performed in one-year old CalpTG and WT mice (Fig. 5). We did not observe any change in arterial blood pressure, renal blood flow or measured glomerular filtration rate at one year (Fig. 5A–C). One year-old CalpTG mice heart weight was 135 ± 9 mg whereas WT mice heart weight was 159 ± 9 mg (n = 7/group, p = 0.05), suggesting that cardiovascular remodelling was ongoing at this time (Fig. 5D).

### Development of aging-related lesions in skin and brain in WT and CalpTG mice

**Figure 6:**
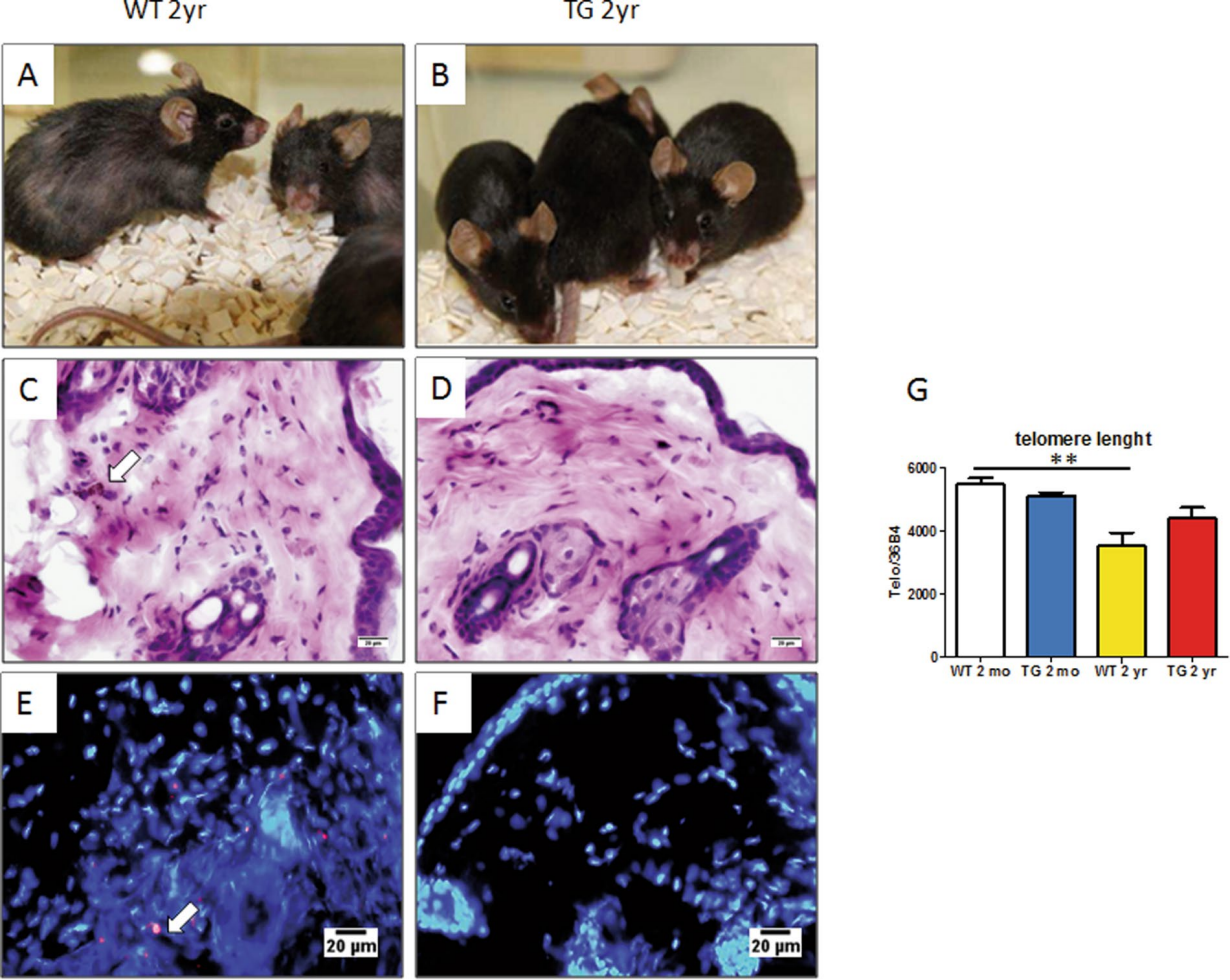
Calpastatin overexpression protects against skin aging. At 2 years, CalpTG (TG) mice (**B**) were phenotypically less impacted by aging than WT mice bred in the same conditions (**A**). There was a macroscopic evidence that CalpTG (TG) mice fur was less affected by aging than WT mice. In addition, we observed less lipofuscin deposits in the skin after hematoxylin eosin staining (**C,D**, white arrow, magnification ×200) or by using lipofuscine autofluorescent properties (**E,F**, white arrow, magnification ×200). Moreover, telomere lenght decreased significantly in WT mice skin but not in CalpTG skin (**G**, **p < 0.01, n = 10/group).

The difference between CalpTG and WT mice in skin phenotype was evident at two years (Fig. 6A,B). There were less lipofuscine deposits, a classical feature of skin aging, in CalpTG mice at 2 years than in WT animals (Fig. 6C–F). In addition, telomere shortening with aging was significant in WT mice but not in CalpTG mice (Fig. 6G, p = 0.0047).

**Figure 7:**
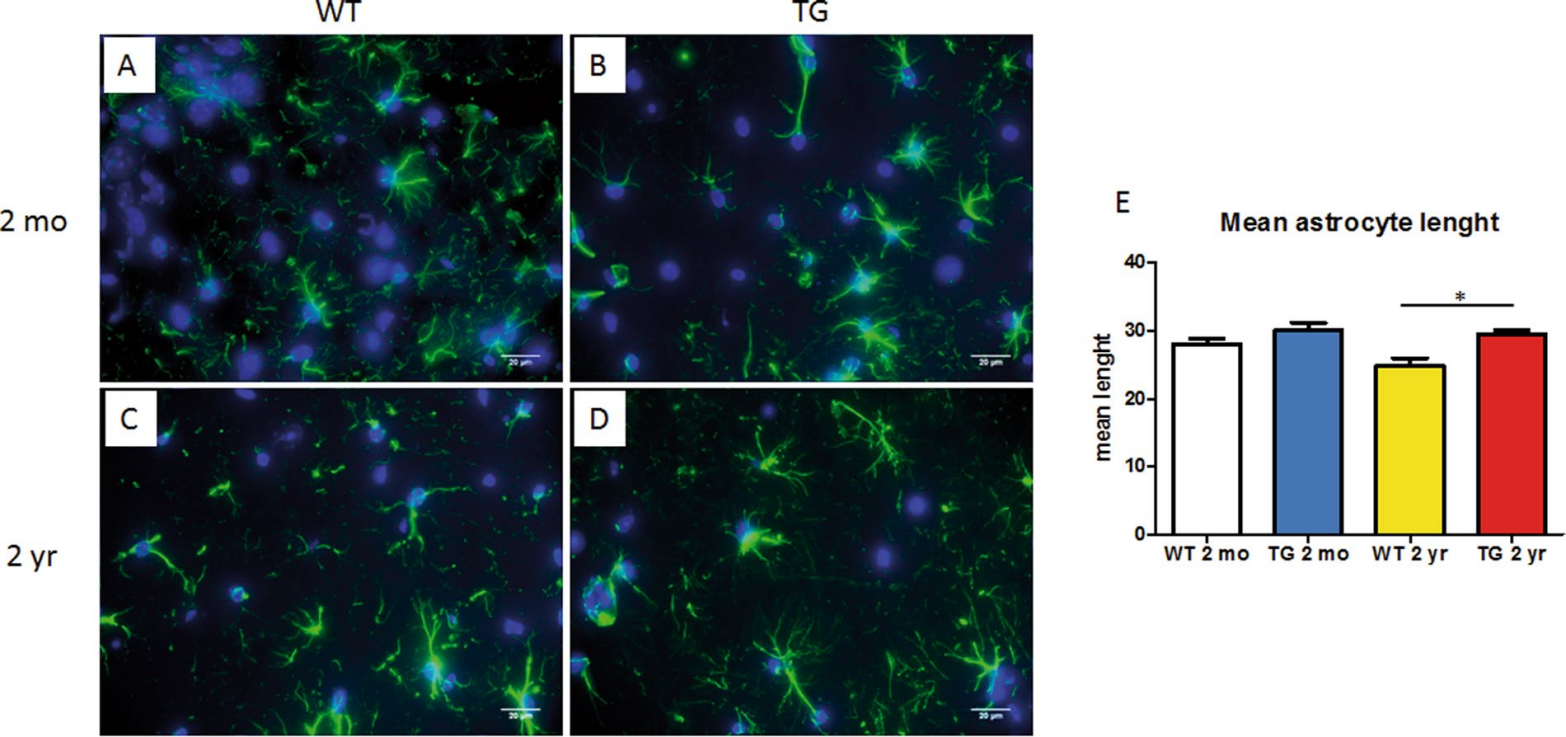
Calpastatin overexpression protects against brain aging. The mean astrocytic lenght decreases with aging in the CA1 area of the hippocampus. This lenght was similar in 2-months old (2 mo) animals (**A,B,E**, magnification ×600). Aged CalpTG (TG) mice were less affected by this decrease than 2 years (2 yr) old WT mice (**C,D,E**, *p < 0.05, n = 6/group, magnification ×600).

Astrocyte process lenght decreases in aged mice brain, especially in the hippocampus CA1 area. CalpTG mice had a relatively preserved mean astrocytic process lenght at 2 years when compared to aged WT mice, confirming the deleterious role of calpains in brain aging (Fig. 7A–E, p = 0.016). We also addressed whether brain blood barrier function would be protected in CalpTG mice by assessment of MECA-32 staining in hippocampus subdivisions, but we did not evidence significant differences between the different groups (not shown).

### Survival and cancer in WT and CalpTG mice

**Figure 8:**
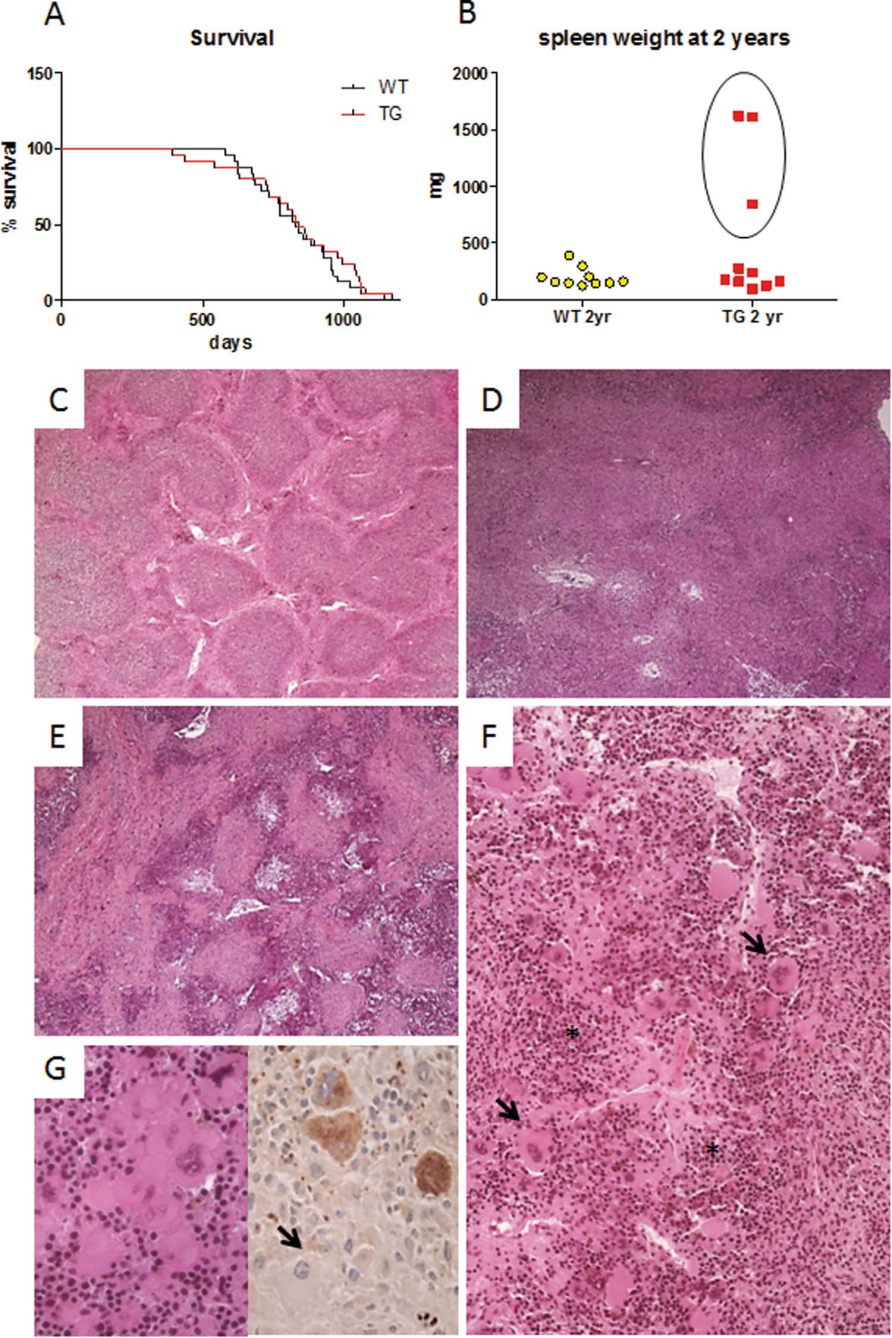
Calpastatin overexpression is associated to an increased frequency of spleen tumors. Most of CalpTG (TG) mice died with an abdominal distension due to a massive splenomegaly, there was no difference of survival rate between WT and CalpTG mice (**A**, n = 25/group). Among the 10 CalpTG mice sacrificed at 2 years, 7 had an apparently normal spleen but 3 were affected by splenic tumors, compressing abdominal organs (**B**). Aged WT mice spleens had a classical follicular architecture and white pulp containing regular lymphocytes (**C**, magnification ×50). Splenic architecture was disrupted in CalpTG tumoral spleens, white pulp could not be differentiated from red pulp (**D**, magnification ×50) and fibrosis was evident in the most severe cases (**E**, magnification ×50). Erythroblastic hyperplasia with evidence of dyserythropoiesis (*), and compact megacaryocytes clusters with nuclear atypia (arrows) were typical features of abnormal myeloid intrasplenic proliferation (**F**, magnification ×200). The partial loss of factor VIII expression by megacaryocytes (arrow) among clusters confirmed the myelodysplasic syndrome (**G**, magnification ×400).

Although CalpTG mice exhibited less aging-related features, the lifespan was not extended in this group (Fig. 8A). Most of CalpTG mice died with an abdominal distension and autopsies revealed voluminous spleens compressing abdominal organs.

Among the 10 CalpTG mice sacrificed at two years to assess aging-related lesions, three had a massive tumoral spleen (Fig. 8B). Histopathological analyses of these tumoral spleens revealed myeloid cell proliferation. Aged WT mice spleens had a preserved follicular architecture and white pulp containing regular lymphocytes, without fibrosis (Fig. 8C). Splenic architecture was disrupted in CalpTG tumoral spleens, white pulp disappeared among red pulp (Fig. 8D) and expansive eosinophilic fibrosis was observed in the most severe cases (Fig. 8E). Erythroblastic hyperplasia with dyserythropoiesis features, and compact megacaryocytes clusters with nuclear atypia evidenced abnormal myeloid intrasplenic proliferation (Fig. 8F). The partial loss of factor VIII expression by megacaryocytes was an hallmark of myelodysplasic syndrome (Fig. 8G). No blastic proliferation has been observed.

### High throughput RNA-sequencing from WT and CalpTG mice renal cortex, differential expression and pathway analysis

**Figure 9:**
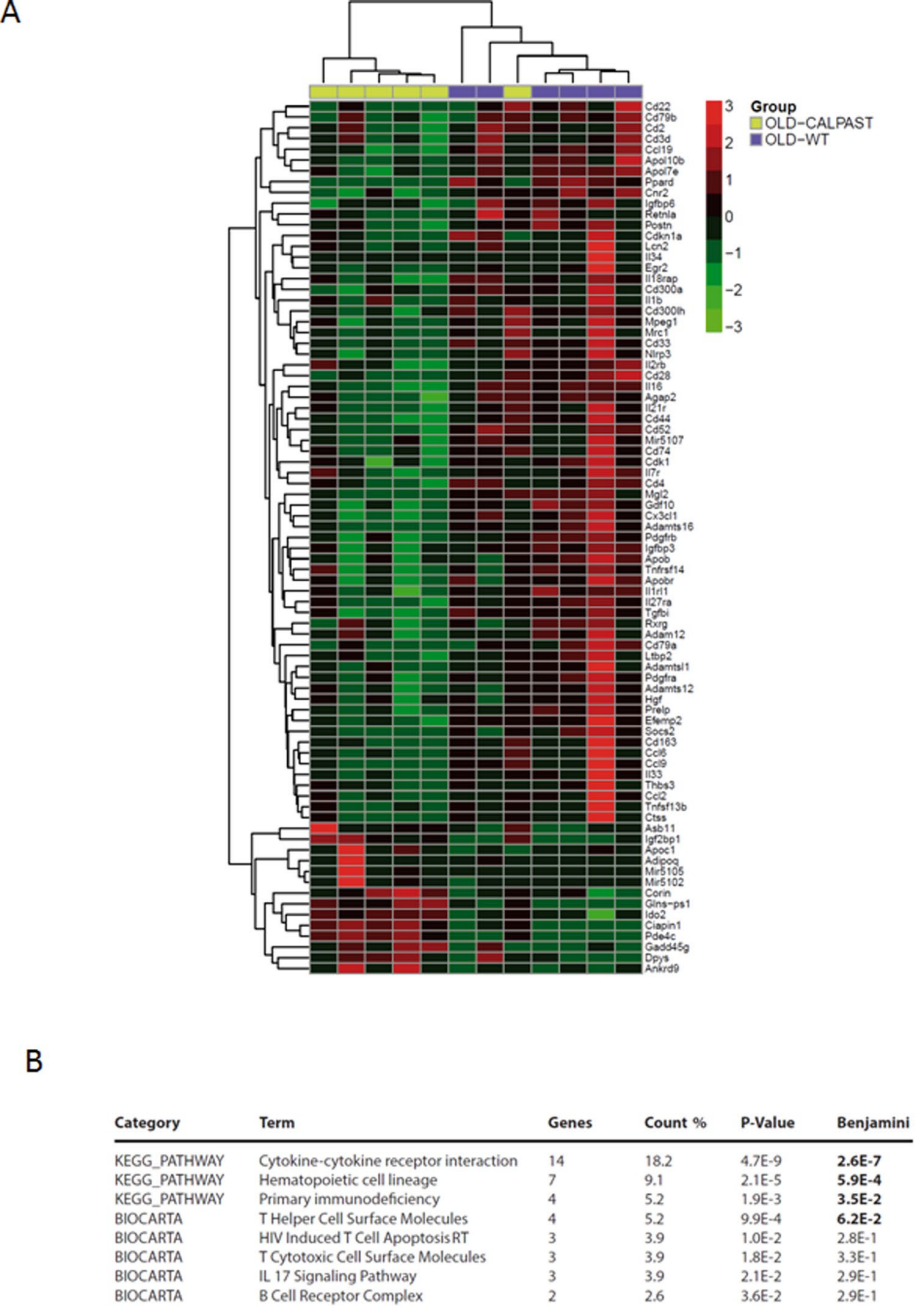
Genes differentially expressed in aged WT and CalpTG mice are related to inflammatory pathways. Heatmap representation of genes differentially expressed between old WT and CalpTG (TG) mice. Eighty-two renal-expressed transcripts have been found significantly differentially expressed between groups. Corresponding genes were represented using the Pheatmap R package, unsupervised clustering, Manhattan method (**A**, n = 6/group). Kegg and biocarta pathways found significantly enriched when comparing old WT and CalpTG mice (**B**). Pathways are immune related suggesting that large parts of difference observed are associated with inflammaging-related genes.

In order to identify the main pathways involved in aging-related lesions, high throughput RNA analyses (RNA-Seq) have been in performed in WT and CalpTG mice renal cortex. Differential expression analysis and pathways analysis revealed that most of differentially expressed pathways were related to immune cell response and that many cytokines involved in innate immunity and particularly in inflammasome activation were differentially regulated between the 2 groups (Fig. 9A,B).

### Development of kidney inflammation with aging in WT and CalpTG mice kidneys

**Figure 10:**
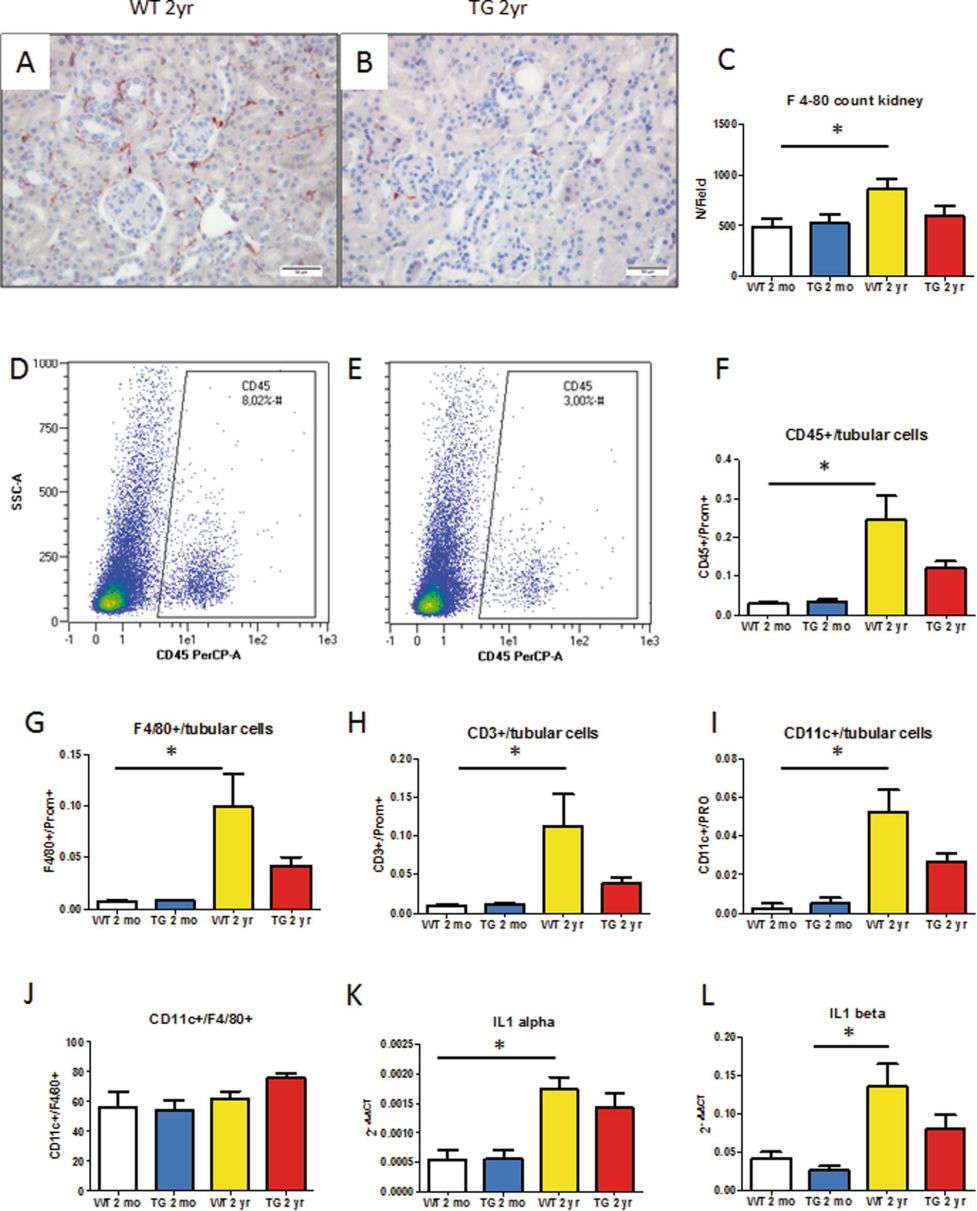
Calpastatin overexpression protects against kidney inflammaging. Kidney infiltration by F4/80+ macrophages was assessed first by immunohistochemistry, evidencing a significant increase with aging in WT mice only (**A–C**, *p < 0.05, n = 8/group, magnification ×400). To improve immune cell quantification, tissue flow cytometry has been performed in another set of experiments to assess the CD45+ (immune cells)/prominin1+ (proximal tubular cells) ratio, evidencing that immune cell infiltrate increased significantly in WT mice only (**D–F**, *p < 0.05, n = 4–6/group). The proportion of macrophages, including M1 CD11+ macrophages, and lymphocytes was significantly increased in old WT mice kidneys only (**G,H,I** *p < 0.05, n = 4–6/group). Nevertheless, the proportion of M1 macrophages was similar in both WT and CalpTG kidneys (**J**, n = 4–6/group). Il-1α expression was very faint in kidney tissue but significantly higher at 2 years in WT mice kidneys when compared to young mice kidneys (**K**, *p < 0.05, n = 6/group). Il-1β was quantitatively more expressed but its expression differed significantly only between aged WT and young CalpTG (TG) (L, *p < 0.05, n = 6/group).

According to RNA-Seq results, further analyses were conducted to compare immune cell infiltrate and cytokine expression in WT and CalpTG mice. Immunohistochemistry evidenced that macrophage infiltration of the renal tissue increased with aging in WT mice but not in TG mice (Fig. 10A–C, p = 0.028). Since immune cell infiltrate was heterogeneous, tissue flow cytometry experiments have been performed in another set of aged and young CalpTG and WT mice to count immune cells and to identify whether macrophages polarization would be influenced by calpains. The proportion of CD45+ immune cells/prominin 1+ proximal tubular cells increased significantly with aging in WT mice but not in CalpTG mice (Fig. 10D–F, p < 0.05). The number of macrophages and lymphocytes infiltrating kidney tissue was significantly increased with aging in WT mice only (Fig. 10G,H, p < 0.05). The proportion of CD11c positive (M1) macrophages was similar in both groups, suggesting that calpain inhibition did not modify macrophages polarization (Fig. 10I,J). The proportion of M2 macrophages (CD206+) was too low to be quantified appropriately (not shown). At last, we confirmed that the expression of cytokines related to inflammasome activation and the main alarmins IL1-α or IL-1β increased with aging in kidneys of WT but not CalpTG mice (Fig. 10K,L, p < 0.05).

### Synthesis of IL-α and IL-1β by WT and CalpTG macrophages ***in vitro*** and *in vivo*

**Figure 11:**
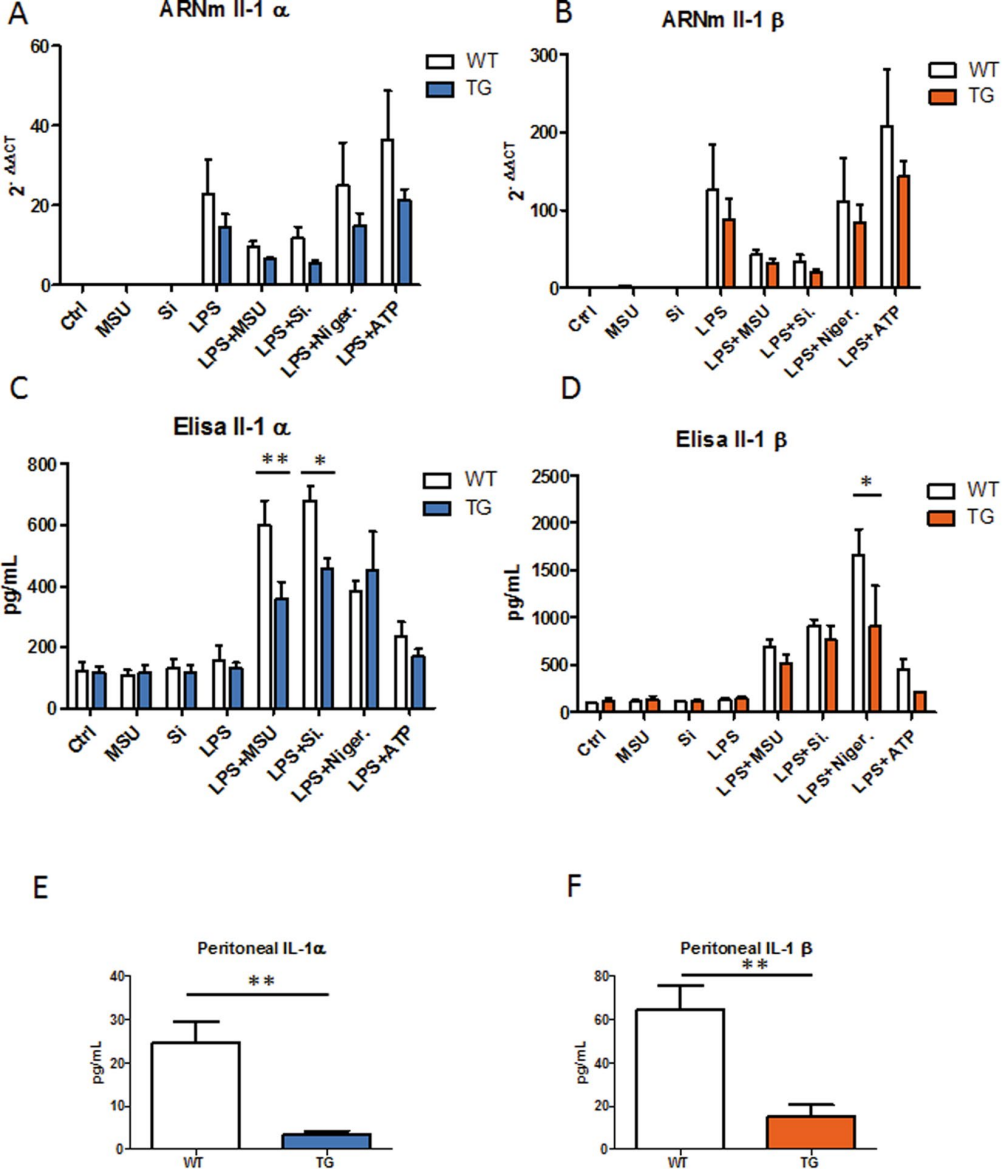
Calpastatin overexpression inhibits IL-1α and Il-1β synthesis in vitro and *in vivo*. *In vitro*, macrophages isolated from WT and CalpTG (TG) mice bones have been exposed to sodium urate crystals (MSU), Silica crystals (Si.), Adenosine TriPhosphate (ATP) or Nigericin (Niger.), with or without priming by Lipopolysaccharides (LPS). IL-1α and IL-1β expression at the mRNA level was globally reduced (ANOVA) by calpastatin overexpression (**A,B**, n = 4/condition). Similarly, IL-1α and IL-1β cytokine production by macrophages was also globally reduced by calpastatin overexpression, especially IL-1α synthesis after exposure to particulate inflammasome activators MSU and Si. (**C,D**, *p < 0.05, **p < 0.01 (post test), n = 4/condition). *In vivo*, MSU crystals have been injected to WT and CalpTG (TG) mice intraperitonally. IL-1α and IL-1β cytokines have been measured early (3 hours) in the peritoneal fluid, their synthesis was dramatically reduced in CalpTG mice (**E,F**, **p < 0.01, n = 5/group).

To assess whether calpain inhibition would affect the synthesis of IL-1α and IL-1β alarmins, the two main cytokines related to NLRP3 inflammasome activation and inflammaging, complementary analyses were conducted *in vitro* and *in vivo*. First, bone-marrow derived macrophages were isolated. We observed that the synthesis of IL-1α and IL-1 β at the mRNA level was globally reduced by calpastatin in response to all inflammasome activators tested (Fig. 11A,B, p = 0.01 and p = 0.05 respectively, ANOVA). Specific calpain inhibition decreased IL-1β concentration in the extracellular milieu in reponse to both particulate and non-particulate inflammasome activators (Fig. 11D, p = 0.003, ANOVA). Of notice, IL-α synthesis was also decreased by calpain inhibition but specifically after macrophage exposure to sodium urate and silica crystals, particulate activators of inflammasome (Fig. 11C, p = 0.008). In addition, we assessed IL-α and IL-1β synthesis at an early phase in a monosodium urate (MSU) peritonitis model. CalpTG mice had lower expression of both IL-1α and IL-1β cytokines, consistent with the *in vitro* experiments and *in vivo* observations in aged mice (Fig. 11E,F, p = 0.008).

## Discussion

The specific inhibition of calpains by calpastatin overexpression protected CalpTG mice against aging-related lesions, especially vascular and kidney lesions. Calpain inhibition reduced kidney inflammaging as evidenced by RNAseq analyses, tissular flow cytometry, immunohistochemistry and complementary *in vitro* and *in vivo* models. To our knowledge, this study is the first to identify calpains as a potent inflammaging mediator.

A protective role of calpain inhibition against aging-related processes has been previously demonstrated previously in neurological disorders. Actually, synthetic calpain inhibitors improved memory and synaptic transmission in a mouse model of Alzheimer disease, and specific calpain inhibition by calpastatin prevented neurodegeneration and restored normal lifespan in tau P301L mice, possibly by limiting the toxic forms of tau^18, 19^.

We have previously demonstrated that specific calpain inhibition reduced inflammation, cardiovascular and kidney lesions induced by angiotensin II infusion^10^. CalpTG mice had a reduced response to angiotensin II signalling and impaired NF-κB activation in kidney and heart tissue^10^. Scalia *et al*. evidenced that calpains are involved in the endothelial adhesion molecule expression in response to angiotensin II/AT1R signalling through the degradation of I-κB and, hence, the upregulation of NF-κB^20^. Furthermore, a pivotal role for calpains in mediating angiotensin II-induced atherosclerosis has been demonstrated^21^. Overexpression of calpastatin in bone marrow-derived cells attenuated significantly angiotensin-II induced inflammation and suppressed macrophage migration and adhesion properties. These studies evidence that the renin angiotensin system (RAS) promotes kidney and vascular lesions through a calpain dependent mobilisation of inflammatory cells, independently from alteration of blood pressure^22^.

During the past years, several lines of evidence argued for an important role of RAS and especially angiotensin II in the development of aging lesions, especially in kidney and arteries^12^. Genetic disruption of the AT1a receptor prevented aging-related progression of cardiac hypertrophy and fibrosis, and extended mouse lifespan^23^. To a lesser extent, RAS pharmacological inhibition was also able to protect against aging related lesions in murine models^24, 25^. Interestingly, we observed that calpain inhibition decreased features of aging particularly in the kidney and the cardiovascular system. Specific calpain inhibition was associated to less cardiovascular hypertrophy and remodelling already present at one year, independently from changes in hemodynamic features and blood pressure at that time. Kidneys from CalpTG mice exhibited less fibrosis and less glomerular lesions. Senescence markers including p21 and beta-galactosidase activity were dramatically lower in CalpTG mice cells, indicating that oxidative stress was reduced. Genome-wide gene expression analyses in 2-years old mice highlighted that inflammation-related pathways differed dramatically between the 2 groups, particularly transcripts of genes involved in innate immunity, NLRP3 inflammasome formation and NF-κB activation. We confirmed that immune cell and especially macrophage infiltration was reduced in CalpTG mice kidneys. There was no difference in M1/M2 polarization, suggesting that inflammatory cell recruitment was influenced by the calpain system but not macrophage polarization.

We have previously described that CalpTG mice were protected against inflammation in models of glomerulonephritis, sepsis or allograft rejection but this is the first demonstration that blunting calpain activity protects against inflammaging^2, 3, 11^. Interestingly, calpain-induced inflammatory processes and classical inflammaging features share similarities. Inflammaging is characterized by a low grade of chronic and systemic inflammation in aging, in the absence of systemic infection or inflammatory disease^15^. One of the main mechanisms involved in inflammaging onset is the NLRP3 inflammasome activation by mitochondrial reactive oxygen species. NLRP3 is a multiproteic complex activating caspase 1, IL-1α, IL-1β and IL-18 processing and synthesis^26^. The synthesis of IL-1α and IL-1β mRNA in response to various NLRP3 inflammasome activators were reduced in CalpTG macrophages. Of notice, the production of IL-1α protein was impacted by calpain inhibition in response to the particulate inflammasome activators (MSU and silica) but much lower in response to ATP or nigericin. This observation is in accordance with the observation by Gross *et al*. that particulate activators, but not nigericin and ATP, are able to induce IL-1α secretion in a way partly independent of the inflammasome, but involving calcium influx and calpain activation^27^.

There is a complex feedback loop between senescence and inflammation. The observation that tubular cells isolated from CalpTG mice are not protected against senescence *in vitro* suggests that calpains promote first inflammation, which then leads to cellular senescence but we cannot rule out that *in vivo*, senescent cells participate in the vicious circle promoting inflammation. Cellular senescence is characterized by the secretion of proinflammatory cytokines (senescence associated secretory phenotype or SASP) including IL-1α, IL-1β, IL-6 or TNF-alpha. NF-κB regulates the majority of genes that comprise the SASP and NF-κB has been demonstrated to drive aging in brain and to be required to enforce many features of aging in a tissue specific manner^28–30^. We and other groups have previously evidenced that NF-κB nuclear translocation and activity was reduced in cells and tissues from CalpTG mice^10, 22^. Here, genome-wide gene expression profiling revealed that cytokines whose synthesis is regulated by NF-κB and that are involved in innate immune response were effectively down-regulated in old CalpTG mice in comparison to old controls.

Although IL-1α is a potent cytokine whose role in kidney lesions and inflammaging is emerging, its expression is much lower than IL-1β expression in vivo and difficult to assess^31^. The *in vivo* model of MSU-induced peritonitis allowed to evidence that both IL-1α and IL-1β production was reduced at an early phase in CalpTG mice, suggesting that calpains actually promote IL-1α and IL-1β synthesis in response to cellular stress *in vivo*.

The main limitation of our study is that despite clearly reduced features of aging in CalpTG mice, lifespan did not significantly differ between the 2 groups. This was due to an increased frequency of spleen tumors in calpTG mice, due to myeloid cell proliferation and fibrosis. We have previously highlighted that calpain inhibition would exert both pro and antitumoral effects in CalpTG mice^32^. In addition, we have evidenced that splenocytes from CalpTG mice have an increased proliferative response *in vitro* ^11^. This is a specific feature of splenocytes since calpain inhibition is usually known to decrease (or have no impact) on cell proliferation^1, 5^. We demonstrated that calpain inhibition amplifies IL-2 signalling via the stabilization of the IL-2 receptor γ-c common chain, providing an explanation for the proliferation response^11^. Interestingly, the γ-c common chain is required to activate JAK3 kinase, which has been implicated in the development of meloproliferative disorders^33^. One may therefore hypothesize that sustained γ-c common chain signalling would in turn promote myeloproliferative disorders. This hypothesis requires further studies including bone marrow analyses.

At last, it would be of interest to determine whether kidney-specific overexpression of calpastatin is sufficient to protect mice against kidney aging or whether its overexpression in immune cells (especially macrophages) is required to limit inflammaging.

As a conclusion, we have evidenced that calpains are a key mediator of inflammaging, especially in the kidney tissue, and that their long-term specific inhibition decreases the impact of aging, markers of senescence and inflammaging mediators. Among the mechanisms involved, SASP-related cytokines production is clearly impacted by calpain inhibition. These results highlight the deleterious role of inflammaging in kidney and vascular lesions occurring with aging.

## Material and Methods

### Mice

Studies were conducted in female mice overexpressing rabbit calpastatin (CalpTG) and control mice (WT) on a C57/bl6 background, bred and housed in similar conditions in a pathogen free zone. These mice were created and characterized in our laboratory. All procedures involving mice were conducted in accordance with national guidelines, institutional policies, local ethical committee and Research Ministry. The experimental protocol was approved by the Charles Darwin Ethical committee and validated by the French research Ministry (Authorization number: Ce5/2012/00686.01).

Mice were housed in constant temperature room with a 12 h dark/light cycle and fed *ad libitum* on standard mouse chow. In a first study, 35 CalpTG and 35 WT mice have been bred and housed together. At two years, 10 mice from each group have been randomized and sacrificed to perform tissue analysis. The 50 other mice were used to perform a survival study. Some of these mice, in the calpTG group, developed distorted abdomen due to splenic lymphoma and were about to die: these animals have been sacrificed in accordance with ethical guidelines but were not excluded from the survival analyses. All other mice participating in the survival study died spontaneously. Ten CalpTG and 10 WT mice bred in similar conditions have been sacrificed at 2 months (“young” groups). In a second step, 10 CalpTG and 10 WT female mice have been bred and housed in similar conditions for one year to perform hemodynamic studies at 12 months. At last, a specific set of 6 CalpTG and 6 WT female mice has been bred together and sacrificed at 2 years to perform kidney flow cytometry tissue analysis, and four 2-months old CalpTG and WT mice have been used as “young” controls.

### Monosodium urate (MSU) induced peritonitis model

MSU crystals were prepared with 500 ml of boiling water and 2 g of uric acid (U2625, Sigma Aldrich). pH solution was maintained at 8 by adding NaOH (1 M). Solution was cooled and kept 24 hours during crystals formation. Crystals were filtered in 100 µm sieve before washing in ethanol and warm sterilization. Peritonitis was induced in 8-weeks old WT and CalpTG females by intraperitoneal (I.P.) injection of 3 mg MSU diluted in 0.5 ml PBS. After 3 hours, peritoneal cavity was flushed with 3 ml PBS heparin for 3 mn. Peritoneal fluid was centrifugated at 4 °C and supernatant has been analyzed by ELISA for Interleukine 1 α (Il-1α) and β (Il-1β) (MLA00, MLB00C, R&D systems).

### Renal tubular cell culture

Kidney fragments from WT or CalpTG mouse have been incubated in 1 mg/ml collagenase 1 solution (Gibco, Life technologies) for 3 mn at 37 °C. Tissue was passed in 100 µm and 40 µm sieves to collect renal tubular cells. Cells were grown in a specific medium to promote tubular cells growth and differentiation containing HAM’s F12 and DMEM medium, insulin 5 µg/ml (I1882, Sigma), dexamethasone 5.10^−8^ M (D8893, Sigma), selenium 60 nM (S913, Sigma), transferrin 5 µg/mL (T1428, Sigma), triiodothyronine 10^−9^ M (T5516, Sigma), EGF from mouse 10 ng/mL (E4127, Sigma), HEPES 20 mM (15630-056, GIBCO), Glutamine 2 mM (25030024, Gibco), 2% Fetal calf serum (Invitrogen), and 0.5% D-Glucose (Sigma).

### Bone marrow derived macrophages (BMDM)

Bone marrow cells from tibia, femur and humerus of donor mice (WT or CalpTG) were obtained after bones were flushed with 1 ml of PBS. Cells were passed through 70 µm sieve, treated with ACK and finally incubated for 7 to 10 days at 37 °C in a medium containing HAM’s F12 250 ml, DMEM 250 ml, decomplemented fetal calf serum 10%, glutamine 200 mmol.l^−1^ and mouse recombinant M-CSF 10 ng.ml^−1^ (ML 416, R&D systems) to obtain adherent BMDM. BMDM were plated in 96 wells (5.10^5^.ml^−1^) and primed with 10 ng.ml^−1^ Lipopolysaccharides (LPS) from Escherichia coli 055:B5 (Sigma-Aldrich) during 3 hours and treated with inflammasome activators for 1 to 6 hours: MSU 300 µg.ml^−1^ (Invivogen), silica particles 100 µg.ml^−1^ (Invivogen), ATP 5 mmol.l^−1^ (Sigma-Aldrich) and Nigericin 5 µmol.l^−1^ (Invivogen). Il-1α and Il-1β excretions were measured in cell supernatant by ELISA (MLA00, MLB00C, R&D systems). Cells mRNA has been collected using the RNeasy kit (Qiagen) according to manufacturer’s instructions.

### Histochemistry and morphometric analyses

Kidneys, heart, aorta, skin and spleen from mice of each group were immersed in AFA and formalin (formaldehyde 4%) solution or snap-frozen. After 24 h in formalin solution, brains have been immersed in 20% sucrose solution for 24 hours and then frozen at −80 °C. Formalin-fixed tissues were embedded in paraffin after conventional processing (alcohol dehydration), and 4-µm thick sections were stained with Masson trichromic solution, hematoxylin-eosin or sirius red in picric acid solution. Heart nuclei were stained with DAPI (Invitrogen, Thermofischer scientific, 1/2000) and cell count/surface was performed to assess myocardic hypertrophy by using an Image J software-based Macro. Perpendicular cross-sections at standardized distance from the renal hilus (0.3 mm) have been performed to measure kidney interlobar arteries media surface. Similarly, four sections of the thoracic descending aorta have been performed to assess the mean media surface for each animal. The number of glomeruli/field, the surface of all glomeruli, and urinary chamber have been measured in 10 renal cortex Masson-stained sections at 200x magnification. For each animal, the mean value has been considered. Kidney fibrosis has been assessed by measuring sirius red-stained surface under polarized light in 10 renal cortex sections at 200x magnification. For each animal, the mean value has been considered. All measures have been performed by using AnalySIS® software.

### Beta-galactosidase activity

β-Galactosidase-related staining was performed on 10 µm unfixed kidney sections with Senescence β-Galactosidase Staining Kit accpording to manufacturer’s instructions (Cell signaling technology, #9860). Stained surface has been measured in 8 renal cortex sections at 200x magnification. For each animal, the mean value has been considered. All measures have been performed by using AnalySIS® software.

### Immunohistochemistry and immunofluorescence

Four-micrometer-thick sections of paraffin-embedded kidneys were dewaxed, heated in citric acid solution and next incubated with antibodies. After blockade of endogenous peroxidase, sections were immunostained with a rat anti-F4/80 monoclonal antibody (MCA497GA, ABD Serotec, 1/2000). The mean cell count was performed on 10 sections at 400x magnification. Four-micrometer-thick cryostat sections were fixed with acetone for 7 min. After blockade of endogenous peroxidase, sections were stained with a mouse anti-CD3 monoclonal antibody (A0452, Dako, 1/200). The mean cell count was performed on 10 sections at 400x magnification. Immunostaining was revealed by specific Histofin (Nichirei Biosciences) and AEC (k34769, Dako) and counterstaining was performed with hematoxylin QS (Vector). An aqueous mounting media was used (Scytek laboratory).

Brains were cut into 10 µm coronal sections after spotting of hypothalamus. Sections were rehydrated and then heated in pH6 citric acid bath. After blocking and permeabilization with PBS BSA 2% and Triton 0.1%, astrocytes were marked with a rabbit anti-GFAP polyclonal antibody (ab7260, Abcam, 1/2000), and secondary antibody Alexa Fluor 488 goat anti rabbit (A11008, Invitrogen, 1/1000). Nucleus was marked with DAPI (Invitrogen, Thermofischer scientific, 1/2000). Astrocytic process measures were performed on 6 stacks of images of hippocampus CA1 region for each mouse. Each stack was obtained by addition of photos at 600x magnification by using an Olympus ix83 microscope and CellSens Dimension software. Astrocytes morphometry was carried out with Image J software, using the plugin NeuronJ. The mean length of astrocytes processes was calculated for each mouse.

Spleens were frozen and secondarily fixed with formaldehyde acetic acid solution. Three-µm sections were stained with hematoxylin-eosin-safran coloration. Megacaryocytes were stained with a rabbit anti-factor VIII polyclonal antibody (A0082, Dako, 1/500).

Tubular cells in culture were fixed with frozen methanol and permeabilized with PBS plus BSA 2% and triton 0.5% solution. Cells were marked with a rabbit anti-p21 monoclonal antibody (ab109199, Abcam, 1/200).Secondary antibody was Alexa Fluor 488 goat anti rabbit (A11008, Invitrogen, 1/1000). Nucleus was marked with DAPI (Invitrogen thermofischer scientific, 1/2000).

### Autofluorescence

Lipofusin deposits were analyzed in frozen skin tissue 4-micrometer thick sections by using autofluorescence lipofuscin properties at 500–640 nm wavelenghts.

### Western blotting

Proteins from WT and CalpTG tissues were extracted in RIPA lysis buffer and protease inhibitor cocktail (1 µg/ml, Sigma). After homogenization, the lysate was centrifuged at 1000 × g for 1 h and the supernatant was frozen at −80 °C. Protein concentration was measured using the Bradford method. 20 µg of protein was separated by electrophoresis on a Bis-Tris gel 4–12% (Novex, Thermofisher). After proteins were transferred onto PVDF membrane, aspecific sites were blocked in PBS Tween and 5% milk solution before incubation with primary antibody overnight at 4 °C. Membrane was washed, then incubated for one hour with secondary antibody conjugated with peroxidase. ECL (RPN 3222, GE Healthcare life sciences) was applied 5 mn on membrane for chemiluminescent reaction. Reaction was revealed on radiographic films (Films were scanned onGS-800 Calibrated Densitometer) or read in Syngen Pxi imager (Ozyme). Optical densities were measured using the software Image J or with GeneSys software (Ozyme). Primary antibodies used were mouse monoclonal antibody anti-spectrin alpha chain (nonerythroid) (MAB1622, Chemicon Millipore, 1/1000), rabbit polyclonal antibody anti-calpain 1 domain IV, (ab39170, Abcam, 1/4000), rabbit polyclonal antibody anti-calpain 2 amino terminal end domain I (ab 39167, Abcam, 1/2000), rabbit polyclonal antibody anti-GAPDH (G9545, Sigma Aldrich, 1/50000),. Secondary antibodies used were anti mouse IgG antibody (NA931V, GE Healthcare, 1/10 000); anti rabbit IgG F (ab 1) 2 fragment antibody (NA9340V GE Healthcare, 1/4000), goat polyclonal antibody to rabbit Ig Secondary G-H & L (ab 6721, Abcam, 1/5000).

### Renal hemodynamics

One-year old mice were anesthetized by pentobarbital sodium (50–60 mg/kg body weight intraperitoneally; Nembutal; Abbott, Chicago, IL) and moved to a servo-controlled table kept at 37 °C. The left femoral artery was catheterized for measurement of arterial pressure, and a femoral venous catheter was used for infusion of volume replacement. Bovine serum albumin (4.75 g/dl of saline solution) was infused initially at 50 μl/min to replace surgical losses, and then at 10 μl/min for maintenance. Arterial pressure was measured via a pressure transducer in left femoral artery (Statham P23 DB, Gould, Valley View, OH), and renal blood flow was measured by a flowmeter (0.5 v probe; Transonic systems TS420, Ithaca, NY). To assess GFR, continuous injection of FITC-inulin infusion was performed at 1 mg.ml^−1^ and urine and blood collections were performed every 15 mn after equilibration. Inulin was measured by fluorometric method. GFR was calculated by measuring renal clearance of inulin over 3 period of time (formula UxV/P, U: urine concentration of inulin, P: plasma concentration of inulin and V: urine output).

### Tissue flow cytometry

Two kidneys from WT and CalpTG, aged and young, mice were dissociated by using a gentleMACS Dissociator (Miltenyi Biotec). Tissue was then passed through a 30 µm sieve. Cells were platted during 30 mn at 4 °C with mouse Fc Block (Miltenyi Biotec). Antibodies were incubated during one hour at +4 °C before analyses with flow cytometry MACSquant analyser (Miltenyi Biotec). Antibodies used were: mouse anti-CD45 PERCP (130-102-469, Miltenyi Biotec), mouse anti-F4/80 PE (130-102-422, Miltenyi Biotec), mouse anti-CD11c APC(17-0114, eBioscience), mouse anti-CD45 vioblue (130-102-430, Miltenyi Biotec), mouse nti-CD3 Violetfluor 450 (75-0032, Tonbo biosciences), mouse anti-Prominin1 APC (130-092-335, Miltenyi Biotec). Results are expressed as ratio of immune cells/tubular cells (prominin1 positive cells), an internal control allowing to quantify immune cells infiltrate in the whole kidney.

### Quantitative RT-PCR

mRNA from kidney cortex was extracted using the RNeasy kit (Qiagen) according to manufacturer’s instructions. RNA concentration was measured by using NanoDrop1000 spectrophotometer (ThermoScientific). RNA were reverse transcribed using Maxima First Strand cDNA Synthesis Kit (Thermoscientific) and PCR was performed using SYBR green and specific primers on a light cycler 480 (Roche). Expression levels were normalized to the house keeping gene GusB (beta-glucuronidase) or 18 S using lightcycler advanced relative quantification program (Roche). Primers used were from eurogentec: Rabbit Cast s: AGCCAGCAAGTCGCTCAG and as: CCATCTCTTTGCTGATTGGAA, mouse Cast s: TCGCAAGTTGGTGGTACAAG and as: CTCCCCAAACTTGCTGCTT, mouse Calpn1 s: AGTGGAAAGGACCCTGGAGT and as: TCTCGTTCATAGGGGTCCAC, mouse Calpn2 s: TGGCTTCGGCATCTATGAG and as: AAGTTTTTGCCGAGGTGGAT, mouse IL1 alpha s: TTGGTTAAATGACCTGCAACA and as: GAGCGCTCACGAACAGTTG, mouse IL1 beta s: TGTAATGAAAGACGGCACACC and as: TCTTCTTTGGGTATTGCTTGG, mouse p21 var1/2 s: TGCGCTTGGAGTGATAGAAA and as: AACATCTCAGGGCCGAAA, mouse Gusb s: CTCTGGTGGCCTTACCTGAT and as: CAGTTGTTGTCACCTTCACCTC, mouse 18 S s: AAGCATTCTGAAATTGGCTCA and as: GTCTCAATCCAGAATGATCAGGT.

### Measure of telomere length by quantitative PCR and telomerase activity

Genomic DNA was extracted from 25 mg renal cortex, skin, heart or tubular cells on column DNeasy blood and tissue kit (Qiagen). DNA quality was verified on Nanodrop 1000 (ThermoScientific). Amplification of genomic telomeres present in DNA was realized in a Light Cycler 480 (Roche) using SYBR green and 2.5 pmol of each specific probe: s CGGTTTGTTTGGGTTTGGGTTTGGGTTTGGGTTTGGGTT, as GGCTTGCCTTACCCTTACCCTTACCCTTACCCTTACCCT, or of a domestic gene, here 36B4 s ACTGGTCTAGGACCCGAGAAG, as TCCCACCTTGTCTCCAGTCT. Denaturation of 5 mn at 95 °C was followed by 30 cycles of development for heart and kidney extracts or 35 cycles for skin extracts. Every cycle consisted of 3 stages (denaturation 15 seconds in 95 °C, hybridization 20 seconds in 52 °C for telomeres or 56 °C for 36B4, strain 15 seconds in 72 °C). Fluorescence was measured at the end of every cycle. After an exponential phase, obtaining of a tray at the end of PCR allowed to calculate a cycle threshold (Ct) for every target DNA, reflection of their initial quantity. Finally, the specificity of PCR product was verified thanks to the curve of fusion realized at the end of PCR. Telomerase activity in kidney was performed with the Telotaggg PCR ELISA kit according to manufacturer’s instructions (12209136001, Roche).

### High throughput RNA-Sequencing from mouse renal cortex, differential expression and pathway analysis

RNA samples from kidney cortex coming from either old C57BL6J (n = 6) or Calpastatin transgenic (CalpTG) mice (n = 6) of high quality (RNA Integrity Number >6) were processed to create mRNA libraries using TrueSeq reagents (Illumina, san diego, California). Clusters were created for Illumina RNA-Sequencing using a CBot system and sequencing was carried out on 2 lanes of HiSeq. 2000 to produce paired-end reads (75 bases length, 29 to 35 millions of reads per sample) and organized per lane to avoid sequencing batch effects. After demultiplexing, bare-coding removal, quality control, paired-end reads were processed from Fastq Sanger format to compact-reads format and then align with STAR (default parameters) to the mouse reference genome (mGC37/mm9). Data alignment and analysis were performed using the GobyWeb interface using Ensembl version 55. Differential expression was computed using the EdgeR package from bioconductor. Data were also examined visually in the Integrative Genomic Viewer (http://www.broadinstitute.org/igv/) respective of Ensembl Annotation (Ensembl version 55) (Anders *et al*., 2013; Dorff *et al*., 2013; Robinson *et al*., 2010). Normalized gene expression level was measured by counts per millions (CPM). Genes that were significantly differentially expressed (DE) between groups (WT vs. CalpT q < 0.05, Edge R P-value adjusted for multiple testing with the Benjamini Hochberg approach) were next selected by their Fold change |LogFC| > 1.2 to build heatmap (R package Pheatmap) and pathway using David 6.8 available at https://david.ncifcrf.gov/home.jsp.

### Statistical analyses

Data are expressed as mean ± SEM. The results were analyzed by non parametric Mann-Whitney and Kruskal-Wallis with Dunn’s multiple comparison test, with the exception of flow cytometry analyses and interleukin-1 dosages which have been analysed by ANOVA and Bonferroni post tests. Results with p < 0.05 were considered statistically significant.

### Data availability

The datasets generated during and/or analysed during the current study are available from the corresponding author on reasonable request.
